# Assessment of cerebrovascular reserve using acetazolamide brain perfusion SPECT in Moyamoya disease 

**DOI:** 10.22038/aojnmb.2025.89265.1647

**Published:** 2026

**Authors:** Harish Goyal, Dhritiman Chakraborty, Srinivas Ananth Kumar, Somnath Pandey

**Affiliations:** 1 Department of Nuclear Medicine, Jawaharlal Institute of Postgraduate Medical Education Research, Puducherry, India; 2 Department of Nuclear Medicine, Institute of Post Graduate Medical Education and Research, Kolkata, West Bengal, India; 3 Quadra Medical Services Pvt Ltd, Kolkata, West Bengal, India

**Keywords:** Moyamoya disease Acetazolamide, Brain perfusion SPECT Cerebrovascular reserve, Rogg’s classification

## Abstract

**Objective(s):**

Moyamoya disease (MMD) is a rare, progressive steno-occlusive cerebrovascular disorder characterized by impaired cerebral perfusion and an elevated risk of ischemic events. Accurate cerebrovascular reserve (CVR) assessment is crucial for guiding surgical decision-making. This study evaluated the clinical utility of acetazolamide (ACZ)-challenged brain perfusion single-photon emission computed tomography (SPECT) in assessing CVR in patients with MMD.

**Methods:**

We retrospectively analyzed 10 patients (6 males, four females; aged 5–65 years) with angiographically confirmed MMD who underwent baseline and post-ACZ ^99m^Tc-ECD SPECT. Regional perfusion across 12 brain regions per patient was visually graded and classified using Rogg’s criteria (Type I–III) to assess CVR.

**Results:**

At baseline, 78/120 regions showed normal perfusion; post-ACZ, this decreased to 72 regions, with an increase in severe hypoperfusion (from 16 to 26 regions). A total of 44 regions demonstrated improved perfusion following ACZ, indicating preserved reserve. According to Rogg’s classification, 63 regions showed Type I, 13 showed Type II, and 39 showed Type III responses. Three patients had infarcts, with two exhibiting crossed cerebellar diaschisis. In a patient who underwent revascularization, new postoperative perfusion defects developed in regions that corresponded to preoperative Type III responses.

**Conclusion:**

ACZ-challenged SPECT effectively characterizes regional CVR in MMD. Identifying Type II and III responses is a valuable predictor for ischemic vulnerability and guides the selection of surgical candidates.

## Introduction

 Moyamoya disease (MMD) is a rare, chronic, and progressive cerebrovascular disorder characterized by stenosis or occlusion of the terminal portions of the internal carotid arteries and their proximal branches, leading to abnormal collateral networks. This results in the classic "puff of smoke" appearance on cerebral angiography (1, 2). First described by Suzuki and Takaku in 1969 (1), MMD typically affects children and young adults and presents clinically with recurrent ischemic strokes, intracranial hemorrhages, or cognitive deficits (2, 3).

 Assessment of cerebrovascular reserve (CVR) is essential in MMD, as impaired CVR correlates with elevated ischemic risk and helps stratify patients who may benefit from surgical revascularization, such as Encephalo- Duro- Arterio- Myo- Synangiosis (EDAMS) or STA-MCA bypass (4–6). The prognostic value of CVR has been substantiated in long-term outcome studies and is recognized in the 2021 Japanese Guidelines for MMD, which emphasize integrating perfusion imaging in clinical workflows (7, 8).

 While digital subtraction angiography (DSA), computed tomography angiography (CTA), and magnetic resonance angiography (MRA) remain essential for anatomical diagnosis, these modalities do not assess cerebral hemo-dynamics. Functional imaging tools-including positron emission tomography (PET), single-photon emission computed tomography (SPECT), arterial spin labeling (ASL), and Blood oxygenation level-dependent MRI (BOLD-MRI) provide regional quantitative or semi-quantitative assessment of CVR (9–11). Among them, acetazolamide (ACZ)-challenged SPECT remains widely used and validated in various clinical settings (5, 12, 13). ACZ promotes vasodilation by increasing carbon dioxide levels through carbonic anhydrase inhibition, thereby exposing areas with reduced vascular reactivity (4, 13). Limited evidence exists regarding the diagnostic and prognostic value of ACZ-challenged ^99m^Tc-ECD SPECT, particularly in resource-limited settings, and regarding the scope of this modality as a CVR screening tool.

 Rogg’s classification allows standardization of CVR interpretation, supporting clinical application and decision-making (12). Kashyap et al. demonstrated that oral ACZ with ^99m^Tc-ECD SPECT could reliably delineate hypoperfused regions, even in resource-limited settings (14). 

 Further, SPECT/MRA fusion and ASL-based techniques such as pseudocontinuous ASL have enhanced the precision of regional perfusion analysis and surgical planning (15, 16). Comparative studies also show the utility of ASL alongside conventional perfusion imaging (11, 17).

 Although the utility of ACZ-challenged SPECT is well-documented, a research gap persists in correlating these standardized CVR patterns with surgical outcomes in diverse, real-world clinical settings. While many reports validate the imaging technique, further evidence is needed to reinforce its predictive power for individual patients undergoing revascularization. The added value of this study is to help fill this gap by providing focused data from a tertiary care center. 

 This study aimed to (1) characterize regional perfusion pre- and post-ACZ using ^99m^Tc-ECD SPECT; (2) classify CVR responses using standardized criteria; and (3) correlate imaging findings with infarct burden and surgical outcomes in patients undergoing EDAMS. We specifically aimed to demonstrate how preoperative CVR classification directly corresponds with postoperative perfusion changes, thereby strengthening the evidence for using ACZ-SPECT as a practical tool for risk stratification and surgical planning.

## Methods

 This retrospective, single-center observational study was conducted at a tertiary care hospital and included patients who underwent ACZ-challenged brain perfusion SPECT between February 2021 and June 2022. The Institutional Ethics Committee approved the study protocol, which waived the requirement for individual patient consent due to the retrospective nature of the analysis. Patient data was anonymized to ensure confidentiality.

### Patient Population

 Eligible patients were identified by reviewing clinical records and imaging databases.

 Inclusion criteria were: (1) a confirmed diagnosis of MMD via digital subtraction angiography (DSA), computed tomography angiography (CTA), or magnetic resonance angiography (MRA); and (2) availability of both baseline and post-ACZ ^99m^Tc-ECD SPECT studies, including follow-up imaging and outcomes for patients who underwent surgery.

 Exclusion criteria included known contra-indications to acetazolamide (e.g., severe renal or hepatic impairment, known sulfonamide allergy), non-diagnostic image quality, or incomplete scan data.

 Ten patients (6 males, four females; age range 5–65 years; mean age 28 years) met these criteria and were included in the final analysis. 

### SPECT Acquisition Protocol

 All patients underwent two brain perfusion SPECT scans using ^99m^Tc-Ethyl Cysteinate Dimer (^99m^Tc-ECD). The baseline scan was acquired under resting conditions. Patients then received oral ACZ for CVR assessment. ACZ tablets (oral diamox), crushed into powder and were administered at a dose of up to 1,200 mg (14 mg/kg) at least 30 minutes before radiotracer injection. A second SPECT study was acquired 15–25 minutes following intravenous injection up to 740 MBq ^99m^Tc-ECD. 

 Both scans were performed using a dual-head gamma camera equipped with low-energy high-resolution (LEHR) collimators. Acquisition parameters included a 128×128 matrix, 360° rotation, and 3° angular increments. Images were reconstructed using filtered back projection and attenuation correction. All patients underwent two separate brain perfusion SPECT scans, with an appropriate interval between studies of 48-72 hours to allow for sufficient radiotracer decay.

### Image Reconstruction and Analysis

 Images were reconstructed using filtered back projection using a Butterworth filter (critical frequency 0.5 and power 10) with Chang’s method for attenuation correction. All processing and analysis were performed on a dedicated medical imaging workstation (GE Infinia Hawkeye 4) using Brain SPECT software.

 For regional analysis, the brain was automatically segmented into 12 standard anatomical regions (frontal, temporal, parietal, occipital lobes, basal ganglia, and cerebellum per hemisphere) using a predefined anatomical template integrated into the software. This generated semi-quantitative data for each region. To determine the percentage of perfusion reduction, regional tracer uptake was normalized to the mean counts within the contralateral or ipsilateral cerebellum, which served as the reference region.

 Two experienced nuclear medicine physicians, blinded to the clinical data, independently reviewed the images and the semi-quantitative results. Discrepancies were resolved by consensus.

 Perfusion status was categorized based on the percentage reduction compared to the cerebellar reference: normal/preserved, mild hypoperfusion (10–25% reduction), moderate hypoperfusion (25–50% reduction), or severe hypoperfusion (>50% reduction).

 CVR was assessed using Rogg’s classification: Type I (normal CVR: ≥5% perfusion increase post-ACZ), Type II (reduced reserve: <5% increase in a previously hypoperfused area), and Type III (exhausted reserve/steal: no change or decreased perfusion post-ACZ) (12).

### Clinical Data and Outcome Correlation

 Clinical data, including history of infarcts and angiographic findings, were recorded. Evidence of crossed cerebellar diaschisis (CCD) was noted on SPECT images. For the two patients who underwent Encephalo-Duro-Arterio-Myo-Synangiosis, post-operative follow-up SPECT imaging and clinical outcomes were reviewed to correlate with the preoperative CVR findings. Descriptive statistics were used given small sample size.

## Results

###  Patient Characteristics and Overall Findings

 Ten patients (six males, four females; mean age 28 years, range 5–65) were included in the study. The diagnosis of MMD was confirmed via MRA in seven patients, DSA in two, and CTA in one. On a case-basis, eight out of ten patients showed some degree of perfusion abnormality at baseline. Radiologic infarcts were identified in three patients, and two of these also exhibited crossed cerebellar diaschisis on SPECT imaging (Table 1). The decision to proceed with surgery was based on a combination of clinical symptoms, angiographic severity, and significant hemodynamic compromise identified on ACZ-challenged SPECT.

**Table 1:** Patient Demographics and Diagnostic Modalities

**Parameter**	**Value**
Number of patients	10
Age range (years)	5 – 65
Mean age (years)	28
Gender	6 Male, 4 Female
** Diagnostic modality used**	
- MR Angiography (MRA)	7 patients (70%)
- Digital Subtraction Angiography (DSA)	2 patients (20%)
- CT Angiography (CTA)	1 patient (10%)
Infarcts on imaging	3 patients (30%)
Crossed cerebellar diaschisis	2 patients
EDAMS performed	2 patients

### Regional Perfusion and Cerebrovascular Reserve Analysis

 A total of 120 brain regions were analyzed across the ten patients. 

 At baseline, 78 regions (65%) demonstrated normal perfusion. The remaining 42 regions (35%) showed hypoperfusion, categorized as mild in 10 (8.3%), moderate in 16 (13.3%), and severe in 16 (13.3%). The most frequently affected locations were the frontal and parietal lobes.

**Figure 1:**
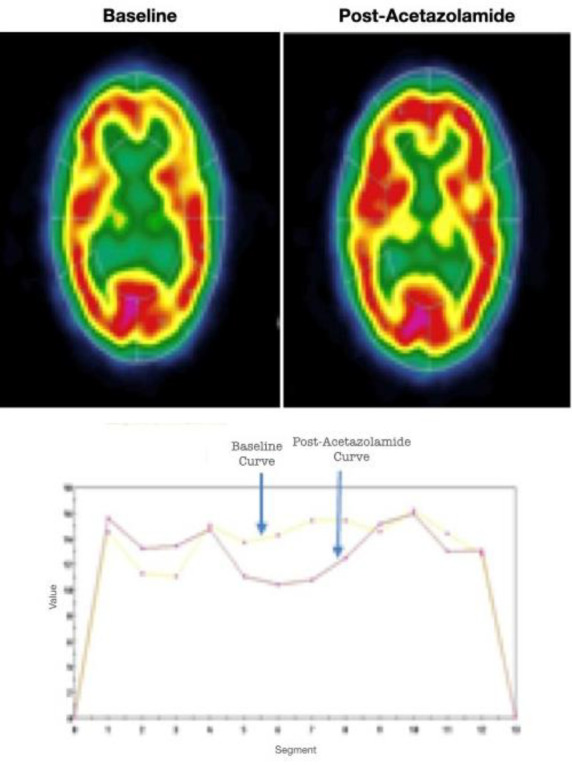
A 24-year-old female with bilateral distal ICA stenosis on MRA underwent ACZ-SPECT for CVR assessment. Post-ACZ images showed improved perfusion in multiple regions, consistent with a Type I response indicating preserved cerebrovascular reserve

 Following the ACZ challenge, the number of regions with severe hypoperfusion increased from 16 to 26 (21.7%), while the number of moderately hypoperfused regions decreased from 16 to 6 (5%). This shift was primarily due to 14 regions showing worsened perfusion, indicating a cerebrovascular steal phenomenon. Despite this, 44 regions (36.7%) showed improved perfusion post-ACZ, indicating some preserved vasoreactivity (Figure 1, Table 2).

**Table 2:** Regional Brain Perfusion Scores (Baseline vs. Post-Acetazolamide)

**Perfusion status**	**Baseline (Pre-ACZ)**	**Post-ACZ**	**Change Observed**
Normal/Preserved	78 regions (65%)	72 regions (60%)	↓ in 6 regions
Mild hypoperfusion	10 regions (8.3%)	16 regions (13.3%)	↑ in 6 regions
Moderate hypoperfusion	16 regions (13.3%)	6 regions (5%)	↓ in 10 regions
Severe hypoperfusion	16 regions (13.3%)	26 regions (21.7%)	↑ in 10 regions
Regions with improved perfusion	—	44 regions (36.7%)	Improved from baseline
Regions with worsened perfusion	—	14 regions (cerebrovascular steal)	Mild/moderate → severe post-ACZ

Note: Total brain regions analyzed = 120 (12 regions ×10 patients)

**Figure 2:**
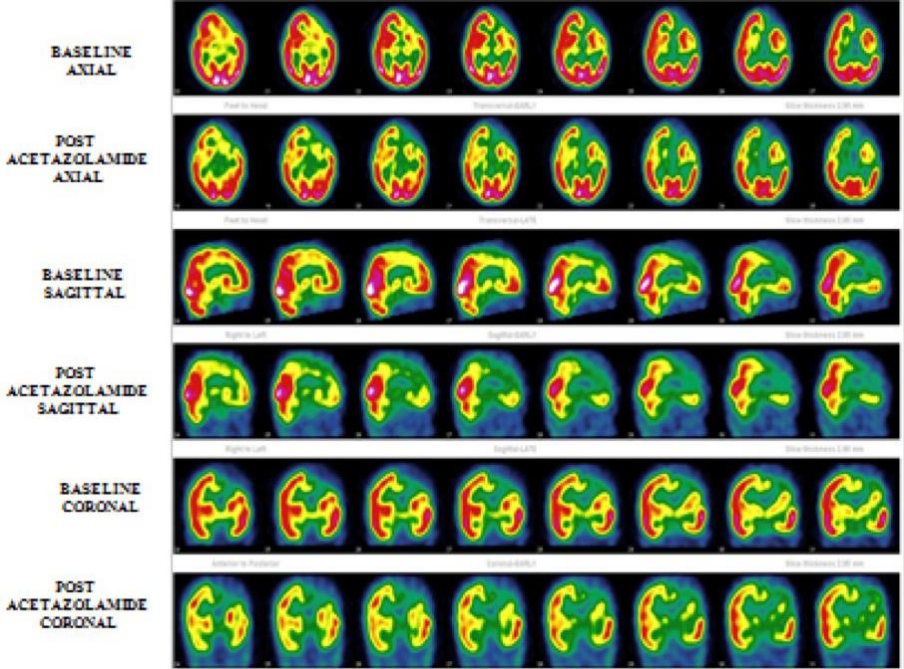
Baseline and post-ACZ SPECT images in an 11-year-old male with recurrent seizures and right hemiparesis show paradoxical perfusion worsening in the left frontal region and other regions post-ACZ, consistent with a Type III response indicating exhausted cerebrovascular reserve

 Using Rogg’s classification, cerebrovascular reserve was categorized across all regions. Type I responses (normal CVR) were present in 63 regions (52.5%). In contrast, evidence of compromised hemodynamics was widespread, with Type II responses (reduced reserve) in 13 regions (10.8%) and Type III responses (exhausted reserve or steal) in 39 regions (32.5%) (Figure 2, Table 3).

**Table 3:** Cerebrovascular Reserve Classification and Perfusion Changes (n=120 regions)

**CVR Type**	**Definition**	**Number of Regions (%)**	**Median Perfusion Change (Range)**
Type I	Preserved reserve	63 (52.5%)	+12% (+5% to +25%)
Type II	Reduced reserve	13 (10.8%)	+2% (-1% to +4.9%)
Type III	Exhausted reserve / Steal	39 (32.5%)	-8% (-2% to -15%)
NC	Not Classified*	5 (4.2%)	N/A

*NC (Not Classified): Refers to regions excluded from CVR analysis due to severe, fixed perfusion defects at baseline (e.g., established infarcts) where a dynamic response could not be reliably assessed

### Surgical Cases and Outcomes

 Two patients with severe hemodynamic compromise underwent EDAMS. The surgical outcomes correlated with preoperative CVR findings.

**Figure 3:**
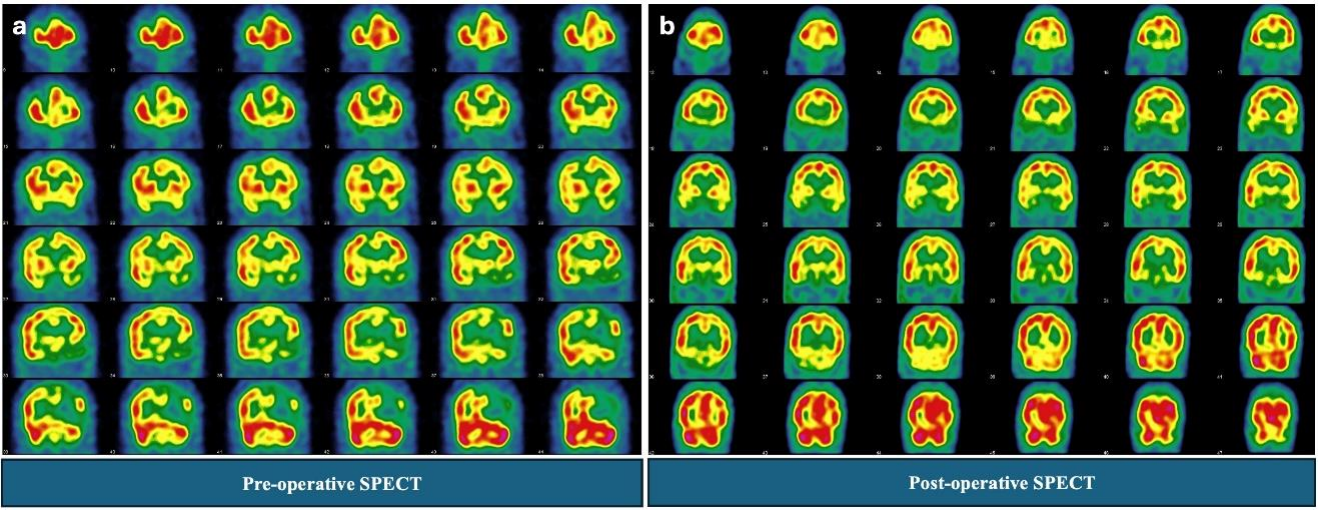
17 years old patient presented with multiple regions of Type II and Type III CVR response in the left hemisphere pre operatively (**a**). Follow-up SPECT performed six months after surgery demonstrated a significant improvement in perfusion to the previously compromised territories, which now showed a normal Type I response (**b**), consistent with successful revascularization and a good clinical outcome

- **Patient 1 (Successful Revascularization):** This patient presented with multiple regions of Type II and Type III CVR response in the left hemisphere preoperatively. Follow-up SPECT performed six months after surgery demonstrated a significant improvement in perfusion to the previously compromised territories, which now showed a normal Type I response, consistent with successful revascularization and a good clinical outcome (Figure 3).

- **Patient 2 (Postoperative Deficits): **

This patient had extensive Type III CVR responses bilaterally in the frontal and parietal regions before surgery. Postoperatively, the patient developed new perfusion deficits.

**Figure 4:**
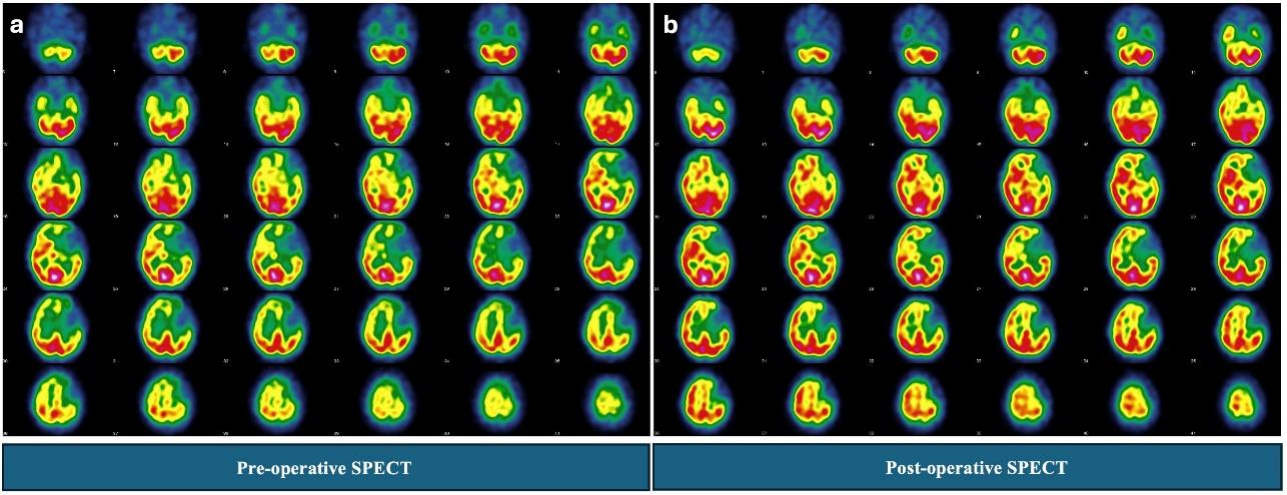
The 29 year old patient had extensive Type III CVR responses bilaterally in the frontal and parietal regions (predominantly left sided) before surgery (**a**). Postoperatively, the patient developed new perfusion deficits. Follow-up SPECT confirmed these new areas of hypoperfusion, which were predominantly located in regions that had demonstrated the most severe preoperative Type III responses (**b**)

 Follow-up SPECT confirmed these new areas of hypo-perfusion, which were predominantly located in regions that had demonstrated the most severe preoperative Type III responses (Figure 4).

## Discussion

 This study demonstrates the feasibility and clinical utility of ACZ-challenged ^99m^Tc-ECD SPECT in evaluating cerebrovascular reserve (CVR) in Moyamoya disease (MMD). We found that a significant portion of assessed brain regions (32.5%) exhibited a Type III response, suggesting exhausted autoregulatory capacity and a high risk of ischemia. These results are consistent with previous reports describing cerebrovascular steal and reduced vasoreactivity in advanced steno-occlusive disease (4, 13).

 A key finding was the increase in severely hypoperfused regions from 16 at baseline to 26 post-ACZ, which underscores the importance of a pharmacologic challenge in unmasking latent hemodynamic compromise that is not apparent on resting scans. These results align with data from Touho et al. and So et al., who also reported the value of CVR measurements for improved surgical planning and prognostication (13, 18). The use of Rogg’s criteria provided a standardized framework for CVR classification, enhancing diagnostic consistency (12).

 Our study also reinforces the value of preoperative CVR evaluation in predicting postoperative outcomes. In the two patients who underwent EDAMS, the postoperative perfusion changes correlated well with the preoperative SPECT findings. The patient with predominantly Type II and III responses who showed postoperative improvement suggests that even compromised tissue can benefit from revascularization. Conversely, the patient who developed new deficits in areas classified as Type III highlights the predictive power of identifying regions with exhausted reserve, which may be more vulnerable to perioperative ischemic events. These observations are in line with reports from Houkin et al. and Cho et al., who found that preoperative hemodynamic status was a key determinant of surgical outcomes (6, 7).

 We also observed crossed cerebellar diaschisis in patients with severe supratentorial infarcts. This phenomenon is characterized by reduced contralateral cerebellar perfusion (hypoperfusion), secondary to interruption of cortico-ponto-cerebellar pathways. The hypoperfusion detected on SPECT reflects diminished perfusion demand in the affected cerebellar hemisphere, a concept originally described by Baron (19, 20).

 This study has several limitations. First, its retrospective design and small sample size restrict the generalizability of the findings. Second, fully quantitative cerebral blood flow measurements and long-term neurocognitive outcome assessments were unavailable, which may have provided additional insights. Third, the absence of standardized quantitative thresholds for CVR assessment across centers limits broader applicability and external validation. Nevertheless, despite these constraints, our results are consistent with prior evidence and support existing national guideline recommendations that emphasize incorporating CVR evaluation into surgical planning for patients with MMD (7).

## Conclusion

 Acetazolamide-challenged SPECT appears to be a valuable tool for assessing CVR in patients with Moyamoya disease. Our preliminary findings, though based on a small cohort, suggest that this technique can help identify regions with hemodynamic compromise and may provide useful information for clinical management. While larger, prospective studies are needed to substantiate the predictive value of this modality, our results support the continued use of functional imaging in the diagnostic workup for this complex cerebrovascular disorder.

## References

[1] Suzuki J, Takaku A (1969). Cerebrovascular "moyamoya" disease Disease showing abnormal net-like vessels in base of brain. Archives of neurology.

[2] Scott RM, Smith ER (2009). Moyamoya disease and Moyamoya syndrome. New England Journal of Medicine..

[3] Kim SK, Cho BK, Phi JH, Lee JY, Chae JH, Kim KJ (2010). Pediatric moyamoya disease: an analysis of 410 consecutive cases. Annals of neurology.

[4] Derdeyn CP, Grubb RL Jr, Powers WJ (1999). Cerebral hemodynamic impairment: methods of measurement and association with stroke risk. Neurology.

[5] Hirano T, Minematsu K, Hasegawa Y, Tanaka Y, Hayashida K, Yamaguchi T (1994). Acetazolamide reactivity on 123I-IMP single photon emission computed tomography in patients with major cerebral artery occlusive disease: correlation with positron emission tomography parameters. Journal of Cerebral Blood Flow & Metabolism.

[6] Houkin K, Kamiyama H, Abe H, Takahashi A, Kuroda S (1996). Surgical therapy for adult moyamoya disease. Can surgical revascularization prevent the recurrence of intracerebral hemorrhage? Stroke.

[7] Cho WS, Kim JE, Kim CH, Ban SP, Kang HS, Son YJ (2014). Long-term outcomes after combined revascularization surgery in adult moyamoya disease. Stroke.

[8] Fujimura M, Tominaga T, Kuroda S, Takahashi JC, Endo H, Ogasawara K (2022). 2021 Japanese guidelines for the management of moyamoya disease: guidelines from the Research Committee on Moyamoya Disease and Japan Stroke Society. Neurologia medico-chirurgica.

[9] Powers WJ, Zazulia AR (2003). The use of positron emission tomography in cerebrovascular disease. Neuroimaging Clinics.

[10] He S, Wang X, Niu H, Liu Z, Zhang J, Hao X (2024). Evaluation of cerebrovascular reactivity in moyamoya disease using oxygen-dependent magnetic resonance imaging. Iscience.

[11] Li J, Jin M, Sun X, Li J, Liu Y, Xi Y (2019). Imaging of moyamoya disease and moyamoya syndrome: current status. Journal of computer assisted tomography.

[12] Rogg J, Rutigliano M, Yonas H, Johnson DW, Pentheny S, Latchaw RE (1989). The acetazolamide challenge: imaging techniques designed to evaluate cerebral blood flow reserve. American Journal of Roentgenology.

[13] Touho H, Karasawa J, Ohnishi H (1996). Preoperative and postoperative evaluation of cerebral perfusion and vasodilatory capacity with 99mTc-HMPAO SPECT and acetazolamide in childhood Moyamoya disease. Stroke.

[14] Kashyap R, Mittal BR, Sunil HV, Bhattacharya A, Singh B, Mukherjee KK (2011). Tc99m-ECD brain SPECT in patients with Moyamoya disease: A reflection of cerebral perfusion status at tissue level in the disease process. Indian Journal of Nuclear Medicine.

[15] Sun W, Ruan Z, Dai X, Li S, Li S, Zhang J (2018). Quantifying hemodynamic changes in moyamoya disease based on two-dimensional cine phase-contrast magnetic resonance imaging and computational fluid dynamics. World Neurosurgery..

[16] Yu Z, Bai X, Zhang Y, Zhang G, Qiu C, Chen L (2022). Baseline hemodynamic impairment and revascularization outcome in newly diagnosed adult moyamoya disease determined by pseudocontinuous arterial spin labeling. World Neurosurgery..

[17] Goetti R, O'Gorman R, Khan N, Kellenberger CJ, Scheer I (2013). Arterial spin labelling MRI for assessment of cerebral perfusion in children with moyamoya disease: comparison with dynamic susceptibility contrast MRI. Neuroradiology.

[18] So Y, Lee HY, Kim SK, Lee JS, Wang KC, Cho BK (2005). Prediction of the clinical outcome of pediatric moyamoya disease with postoperative basal/ acetazolamide stress brain perfusion SPECT after revascularization surgery. Stroke.

[19] Baron JC, Bousser MG, Comar D, Castaigne P (1981). Crossed cerebellar diaschisis in human supratentorial brain infarction. Transactions of the American Neurological Association..

[20] Sin DS, Kim MH, Park SA, Joo MC, Kim MS (2018). Crossed cerebellar diaschisis: risk factors and correlation to functional recovery in intracerebral hemorrhage. Annals of Rehabilitation Medicine.

